# Regenerated Cellulose Films Coated with Waterborne Polyurethane with Enhanced Mechanical Properties

**DOI:** 10.3390/polym17070890

**Published:** 2025-03-26

**Authors:** Renxiang Xiong, Jinping Zhou

**Affiliations:** Hubei Engineering Center of Natural Polymers-Based Medical Materials, College of Chemistry and Molecular Sciences, Wuhan University, Wuhan 430072, China

**Keywords:** cellulose, waterborne polyurethane, surface engineering strategy, wet strength, biodegradability

## Abstract

Regenerated cellulose (RC) films with abundant sources and low processing costs are considered to be excellent biodegradable and recycled packaging materials. However, there is still a problem to be solved: the poor strength of RC films in the wet state. Polyurethane (PU) possesses excellent mechanical properties, biocompatibility and biodegradability. In this work, a PU coating is successfully introduced on the RC film surface via a facile surface engineering strategy, followed by plane hot-pressing process, and the RC@PU films are obtained. Notably, under wet conditions, RC@PU films show outstanding mechanical properties (fracture stress of 22.5 MPa, fracture strain of 75.9%, toughness of 10.6 MJ/m^3^), which are greater than those of the pure RC films (18.9 MPa, 56.5%, 6.9 MJ/m^3^). In addition, RC@PU films play an important role in anti-water evaporation tests. Moreover, RC@PU films exhibit excellent biodegradability, which can be completely degraded in a natural environment in about 70 days. This work provides a simple and feasible surface engineering strategy for developing RC films with excellent wet strength and biodegradability.

## 1. Introduction

With the depletion of fossil fuels and the associated problems, many studies are focusing on the development of bio-based renewable and sustainable materials that have broad prospects [1]. Cellulose, one of the most abundant and widely used renewable biomass sources in the world [2,3], has garnered significant attention due to its hierarchical structure, good biodegradability, and non-toxicity [4,5]. As a result, cellulose-based materials are widely utilized in various fields, including sensors, the paper industry, food production, and packaging [6,7,8,9].

Although cellulose has the advantages of wide raw materials, abundant reserves and good biodegradability, the weak mechanical properties of cellulose films in a wet state greatly limit their application scenarios in the field of ecofriendly packaging materials [10,11]. Therefore, improving the weak mechanical properties of cellulose films under wet conditions is still one of the problems to be solved at present. In previous work, Shen et al. obtained high wet strength fibers by introducing cyanoethyl groups with a degree of substitution of 2.77 into cellulose matrix and wet spinning cyanoethyl cellulose [12]. Additionally, incorporating methylene diphenyl diisocyanate into cellulose filter paper and adding −(CH_3_)_3_N^+^ groups to cellulose and hemicellulose wood films can improve the wet strength of these materials [13,14]. However, the introduction of other functional groups can reduce the biodegradability of cellulose-based materials [15]. Wang et al. separated lignin-containing cellulose nanofibers from tobacco stems using ammonium sulfite cooking and formic acid hydrolysis, subsequently preparing cellulose nanopapers with high wet strength [16]. Lakovaara et al. developed a multifunctional biomaterial with improved wet strength by creating cellulose nanoworm networks from esterified cellulose using an ethanol swelling method [17]. Although these methods have improved the wet strength of cellulose materials, their preparation processes are relatively complex. Therefore, there is a need to develop simpler and more feasible methods to enhance the wet strength of cellulose films.

Polyurethane (PU), being a relatively polar polymer, can interact with the polar groups of cellulose to produce strong interfacial adhesion [8]. Composite materials based on cellulose and PU have been developed to some extent. Pinto et al. prepared the nanocomposites of bacterial cellulose nanofibers (BCN) and polyurethane prepolymer. The interfacial reaction between BCN and PU prepolymer was improved by the solvent exchange process. The nanocomposites have remarkable thermal stability and have the application potential of flexible electronic devices [18]. Liu et al. prepared cellulose/polyurethane composite gel and applied it to a new lithium-ion battery electrolyte, showing excellent electrochemical performance [19]. Stanzione et al. used ball-milled cellulose (Cc) and ultrafine ball-milled amorphous cellulose (Ca) as reactive fillers to adjust the mechanical and thermal properties of polyurethane–cellulose composite foam. PUCa-20, which has the best performance, shows excellent thermal insulation and mechanical properties [20]. In this study, we propose a surface engineering strategy to introduce a PU coating onto the surface of regenerated cellulose (RC) films to enhance their mechanical properties. RC@PU films were prepared by soaking RC films in a waterborne polyurethane (WPU) solution at a specific concentration for 24 h, followed by a hot-pressing process. As a result, a PU coating was formed on the surface of the RC films. We investigated the structures of the RC films and PU, as well as the structure and physicochemical properties of the RC@PU films. The mechanical properties of the RC@PU films are superior to those of the RC films under both wet and dry conditions, and the water evaporation retarding ability and hydrophobicity of the RC@PU films are enhanced. Additionally, the RC@PU films exhibit excellent biodegradability. This work achieves the goal of improving the mechanical properties of RC films in a simple and feasible manner, with potential applications in ecofriendly packaging.

## 2. Materials and Methods

### 2.1. Materials

The viscosity-average molecular weight of cellulose (cotton linter pulp) is M_η_ = 9.5 × 10^4^ g mol^−1^, as determined by using a viscometer in cadoxen at 25 °C. WPU 101F was purchased from Wuhan Hongyi Copolymerization New Material Technology Co., Ltd. (Wuhan, China). LiOH·H_2_O, urea and concentrated sulfuric acid were purchased from Sinopharm Chemical Reagent Co., Ltd. (Shanghai, China). All reagents were used as received without further purification.

### 2.2. Preparation of RC@PU Films

The cellulose was dissolved in 8 wt% LiOH·H_2_O/15 wt% urea aqueous solution to obtain a transparent solution (6 wt%) [21,22]. The cellulose solution was placed at −40 °C until the solution was completely frozen. After being thawed and mechanically stirred at 800 rpm, the cellulose was fully dissolved. The cellulose solution was evenly divided into the centrifugal tube, and then centrifuged (−3 °C, 6000 rpm, 10 min) to remove bubbles. The centrifuged solution was poured onto a glass plate, and the film was spread by the casting method. The gel was regenerated in a 5 wt% sulfuric acid solution, and then washed with deionized water to obtain a regenerated cellulose (RC) hydrogel film. In addition, the RC hydrogel film was soaked in WPU solution for 24 h, then sandwiched between two stainless steel plates, followed by a hot pressing (Qi En Technology, Wuhan, China) at 107 °C, 3 MPa for about 2 h. The RC@PU film was obtained when the film was removed from the hot press plate and the temperature dropped to room temperature (25 °C). According to the concentration of WPU solution (1, 2, 3, 4, 5 wt%), the films were coded as RC@PU1, RC@PU2, RC@PU3, RC@PU4 and RC@PU5, respectively. RC hydrogel film without immersing in WPU solution was coded as RCH, and the RC film obtained after hot pressing without immersing in WPU solution was coded as RC0.

### 2.3. Characterization

Fourier infrared spectra of the films were measured using the attenuated total reflection (ATR) method on a Fourier infrared spectroscopy analyzer (FTIR5700, Thermo Fisher Scientific, Waltham, MA, USA). X-ray diffraction patterns were measured using the reflection pattern of Cu Kα radiation (λ = 0.154 nm) on the Rigaku Miniflex600 diffractometer (Japan). The thermal stability was measured by a TGA2 thermogravimetric analyzer (Mettler-Toledo, Zurich, Switzerland). All samples were placed in Al_2_O_3_ crucibles, and then the temperature was raised from 30 to 800 °C at the rate of 10 K min^−1^ in the N_2_ atmosphere. The transmittance of the samples at 300–800 nm was measured by an ultraviolet-visible spectrophotometer (Evolution 201, Thermo Fisher Scientific, MA, USA).

Scanning electron microscopy (SEM) images of the films were taken with a field emission scanning electron microscope (Zeiss Gemini500, Zeiss, Cambridge, UK) at an accelerated voltage of 3 keV. All samples were frozen in liquid nitrogen and snapped immediately. After the RCH was broken, the RCH was immediately freeze-dried to remove the water and ensure that the cellulose structure was not destroyed. The surface and cross sections of all samples were sprayed with gold. Sectional energy dispersive X-ray spectroscopy (EDS) images of RC@PU1 were performed on a field emission scanning electron microscope (Tescan CLARA, Tescan, South Moravia, Czech Republic) at an accelerated voltage of 5 keV. Surface topography images of RC0 and RC@PU series were obtained by atomic force microscopy (SPM-9700HT, Shimadzu, Kyoto, Japan).

The water contact angles of the samples were measured by a contact angle measuring instrument (KRUSS DAS100S, Kruss-scientific, Hamburg, Germany). A drop of water (1 µL) was dripped to the surface of the film by the instrument and photographed within 10 s. Each sample was tested in parallel three times, and the water contact angles on the surface of the film samples were calculated by Image J software (1.54 g) and averaged. Mechanical properties were measured by an electronic universal material testing machine (Instron 5967, Instron, Norwood, MA, USA). The sample was cut into a rectangle (5 cm × 1 cm). The dried films were stretched at room temperature at a rate of 2.0 mm min^−1^. The films were soaked in deionized water for 24 h to obtain wet samples, which were immediately stretched at room temperature at a rate of 5.0 mm min^−1^. When the film had been stretched and broken, the test was terminated. All samples were repeated at least five times. Young’s modulus was calculated from the initial linear region of the stress–strain curve, and toughness was calculated from the integral area of the stress–strain curve from the initial to the breaking point. The wet resistance (*W_R_*) of the sample is calculated as follows [23]:

(1) $$W_{R}(\%)=\sigma_{wet}/\sigma_{dry}\times 100\%$$

where *σ_wet_* and *σ_dry_* are the tensile strength of the films under wet and dry conditions, respectively.

The particle sizes of PU in different concentrations of WPU solutions were determined by Zetasizer Ultra (Malvern Panalytical, Malvern, UK). The 30 wt% WPU solution was diluted into 1, 2, 3, 4 and 5 wt% WPU solutions, and then poured into a colorimetric dish for testing.

### 2.4. Swelling and Water Evaporation Tests

The film was cut into a rectangle (5 cm × 1 cm). Dry weight (*M*_d_) was weighed after drying the film in a vacuum oven at 100 °C for 5 h, and then the film was immersed in deionized water at 25 °C for 12 h, 24 h and 36 h. Then, the film was removed from the water, gently wiped with filter paper to remove water stains on the surface, and quickly weighed to obtain swelling weight (*M_s_*). Each group of films was tested three times at least. The swelling ratio (*SR*) is calculated as follows [24]:

(2) $$SR\left(\%\right)=\frac{M_{s}-M_{d}}{M_{d}}\times 100\%$$

where *M*_d_ and *M_s_* are the masses of the dry and swelling films, respectively.

The film was cut into rectangles (3 cm × 3 cm). Simple measuring devices for water evaporation rate were prepared by 50 mL centrifugal tubes. The port radius of the centrifuge tube was 1.37 cm. About 16 mL of deionized water was added to each centrifuge tube. The cut film was completely covered with the centrifugal tube mouth, and the excess part of the film connected with the tube mouth was glued to the outer end of the centrifugal tube mouth, so that the inside and outside of the centrifugal tube were only communicated by the film. A simple measuring device was prepared, and the initial mass *M*_0_ of the device was measured on day 0. The simple device was vaporized in a natural environment, and the mass of the device *M_n_* (mass on the Nth day of evaporation) was measured once every day. Each group of films was tested three times in parallel. The weight loss rate (*WLR*) of water for each set of devices is calculated as follows [25]:

(3) $$WLR=\frac{M_{n}-M_{0}}{\pi r^{2}}$$

where *M_n_* and *M*_0_ are the masses on the N days and original state of evaporation of the simple measuring device for water evaporation rate, respectively, and r is the radius of the nozzle of the centrifugal tube.

### 2.5. Biodegradation Test

Six batches of RC0 and RC@PU1-5 films (1.5 cm × 1.5 cm) were wrapped in nylon fabric (500 mesh) and buried in natural soil below 10 cm [26], and the ambient temperature of the test was mainly in the range of 10–30 °C. After 7–70 days of burial, the samples were taken out and dried, and the degradation of the samples was characterized by digital photos. RC@PU2 was weighed each time to obtain the curve of degradation time dependence of weight loss. At the same time, the degradation of RC0, RC@PU2 and RC@PU5 films were characterized by SEM.

### 2.6. In Vitro Cytotoxicity Assay

The cytotoxicity of RC0 and RC@PU2 films to L929 cells was quantitatively evaluated by cell counting kit -8 (CCK-8). L929 cells were first cultured in a 96-well plate at a density of 1 × 10^4^ cells/well for 24 h, and then co-cultured with samples with equivalent DOX concentration of 0.01*–*16 μg/mL. After 24 h of incubation, the medium was removed, washed with PBS, and then 200 μL of DMEM medium containing 10% CCK-8 reagent was added to each well, and incubated at 37 °C for 2 h. Finally, the optical density (OD) of each well was monitored at 450 nm on a microplate reader (Spark 10M, Tecan, Männedorf, Switzerland). The cell viability was estimated as follows [27]:

(4) $$Cell\;viability=\frac{OD\;value\;of\;sample\;group}{OD\;value\;of\;control\;group}\times 100\%$$

## 3. Results and Discussion

### 3.1. Structure and Morphology of RC@PU Films

**Figure 1:**
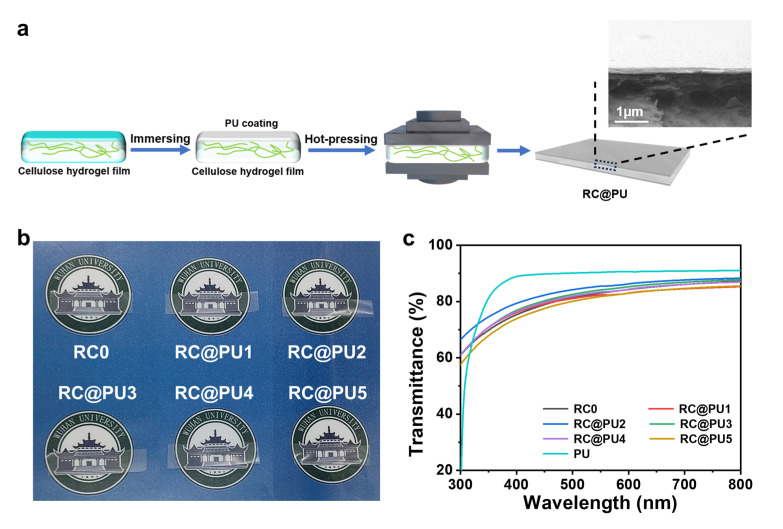
(**a**) Illustration for the preparation of RC@PU films. (**b**) Photographs of RC0 and RC@PU films. (**c**) UV-Vis transmittance of PU, RC0 and RC@PU1-5.

The preparation of RC@PU films is shown in Figure 1. Firstly, cellulose cottons were dissolved in a LiOH/urea aqueous solution. The regenerated cellulose hydrogel (RCH) films were then obtained by regeneration in a 5 wt% sulfuric acid solution, followed by washing with deionized water. The RCH films, which exhibited a loose and porous structure (Figure S1a–d), were beneficial for the subsequent solvent exchange process. These RCH films were then immersed in an aqueous WPU solution with varying contents. Finally, RC@PU films were obtained by hot pressing. Figure 1b shows the light transmission of RC0 and RC@PU series films under natural light, with the “Wuhan University” logo clearly visible. Additionally, the light transmittance of PU, RC0 and RC@PU films was further characterized using a UV-VIS spectrophotometer (Figure 1c). All film samples demonstrated good light transmittance in the visible wavelength range (400–760 nm).

**Figure 2:**
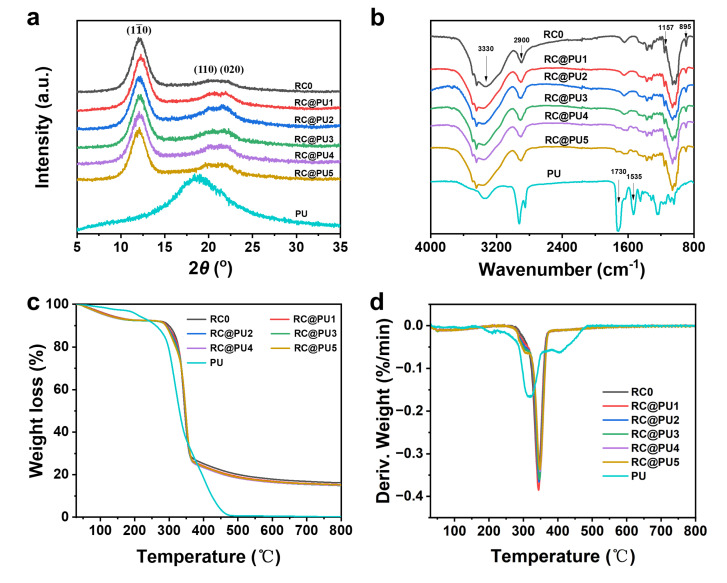
(**a**) XRD patterns, (**b**) ATR-FTIR spectra, (**c**) TG and (**d**) DTG curves of the RC0 and RC@PU films.

Figure 2a shows the XRD patterns of PU and RC@PU films. It can be observed that PU is in an amorphous state, with a broad peak at 2*θ* = 19.0°. The RC0 and RC@PU films exhibit characteristic diffraction peaks of cellulose II crystals at 2*θ* = 12.1°, 20.0° and 21.8°, corresponding to the (1$\bar{1}$0), (110) and (020) crystal planes, respectively [28,29]. This suggests that the introduction of PU does not alter the crystalline structure of cellulose as the main component. The ATR-FTIR spectra of RC0, RC@PU and PU films are shown in Figure 2b. The FTIR spectrum of RC0 shows the characteristic peaks of cellulose at 3330, 2900, 1157, 1059, 1020 and 895 cm^−1^, which correspond to the stretching vibrations of O-H, C-H, C-O-C, and C-OH groups on the glucose ring, as well as the bending vibration of C-O-C [30,31,32]. Additionally, the C-O-C bending vibration absorption peaks of the cellulosic glycoside bond are found in both RC0 and RC@PU films at 895 cm^−1^, indicating that the chemical structure of cellulose in all films remains unchanged during the hot-pressing process. The characteristic absorption peaks of PU are observed in the FTIR spectrum of PU at 3350, 2920, 2850, 1730, 1539, and 1245 cm^−1^, which correspond to the N-H stretching, C-H stretching, and C-H stretching vibrations in ⟩CH-NH-, C=O stretching, C-N stretching, and N-H bending vibrations, respectively [33,34]. Compared with RC0, RC@PU4 and RC@PU5 films show absorption peaks at 1730 cm^−1^ and 1535 cm^−1^, which correspond to C=O and C-N stretching vibrations, respectively. This indicates that PU was successfully incorporated onto the surface of RC films. Furthermore, all the characteristic absorption peaks of RC0 are present in RC@PU films, which further confirms that the cellulose structure in RC@PU films is still intact after the introduction of PU.

In order to evaluate the thermal stability of PU, RC0 and RC@PU films, the thermogravimetric curves of each group of films were obtained by testing, as shown in Figure 2c. The thermal decomposition of PU occurs between 150 and 480 °C, while the thermal decomposition of cellulose mainly occurs between 260 and 370 °C. The thermal decomposition rates of PU, RC0 and RC@PU films during the test are shown in Figure 2d. The thermal decomposition of PU is primarily concentrated around 318 °C. Due to the introduction of PU, a peak in the RC@PU films appears near 300 °C compared to RC0, which is attributed to the decomposition of PU in the RC@PU films. The decomposition peaks of cellulose appear in both RC0 and RC@PU films near 343 °C [35], and their decomposition temperatures are listed in Table S1. The decomposition temperatures of RC@PU films are greater than the decomposition temperature of RC0, indicating that the introduction of PU improves the thermal stability of the cellulose films to some extent. These results further confirm the strong hydrogen bond interactions between PU and cellulose [36].

**Figure 3:**
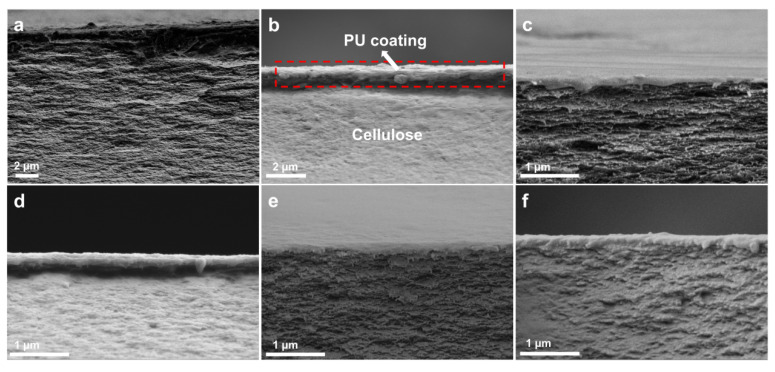
SEM images of the cross-section of (**a**) RC0 and (**b**–**f**) RC@PU1-5 films.

The cross-sectional morphologies of RC0 and RC@PU films are shown in Figure 3, and the SEM image of the cross-section of PU film is shown in Figure S5a. It can be observed that the cross-section of PU film is smooth; meanwhile, the cross sections of RC0 and RC@PU films are characterized by stratification and density. By comparing the RCH section (Figure S1c,d) and the RC film section soaked in 1 wt% PU solution and air-dried (Figure S2b), the formation process of the RC0 and RC@PU films cross-sections could be further understood. The film formation of cellulose in RCH can be better reduced by liquid nitrogen freezing and freeze-drying. The cross-section of RCH is loose and porous, while Figure S2b shows that RC film has a certain degree of stratification, loose and porous cross-section in the state of natural air drying [37,38]. Therefore, the stratification and densification of RC0 and RC@PU film sections can be attributed to the fact that RC films are extruded to the middle under the action of high pressure on both sides during the hot-pressing process, which intensifies the stratification phenomenon, and the spacing between cellulose chain segments decreases, making the films more compact [39]. Compared with RC0 (Figure 3a), a layer of coating substance could be observed on the layered dense cellulose film in the sectional SEM diagram of RC@PU1-5 (Figure 3b–f), and it is reasonable to assume that the coating substance is PU coating. RC@PU1 soaked by immersion solution with minimum PU content is selected for cross-sectional EDS test, and the results are obtained as shown in Figure S3 and Table S2. In the cross-section SEM diagram of RC@PU1 (Figure S3a), three points (b, c and d) are selected from top to bottom for EDS point scanning. Among them, point b is located at the coated material, point c is located at the junction between the coating and the cellulose film, and point d is located on the cellulose film. In the EDS images, there is an obvious N-peak at point b, a weak N-peak at point c, and a little N-peak at point d. In the element content table of each point obtained by the test (Table S2), the mass fraction (ω) of N at point b is 1.07 wt%, and ω (N) > 3 × N_σ_, which proves that there is N element at point b. The mass fraction of N at point c is 0.48 wt%, but it does not meet the condition of ω (N) > 3 × N_σ_, which indicates that there may be N elements at point c. The mass fraction of N at point d is 0, suggesting that point d does not contain N elements. Since the N element existed only in PU in RC@PU films, the coating substances in RC@PU films cross-section are indeed PU, and PU existed in RC@PU films essentially in the form of coatings on the surface of cellulose films. At the same time, it is further proved that after soaking and hot-pressing steps, PU was successfully introduced and retained on the RC@PU films in the form of coatings. In Figure S2b, the thickness of the PU coating on the RC@PU film soaked in 1 wt% PU solution and naturally air-dried is about 1 µm. By measuring the coating thickness in multiple SEM images of each group of RC@PU films, the coating thickness curve of RC@PU1-5 is obtained (Figure S4). The coating thickness of RC@PU films ranges from 0.17 to 0.27 µm, and the coating thickness increases with an increase in PU content in the immersion solution. By comparing the coating thickness before and after hot pressing, it can be found that the PU coatings on RC@PU films also become more compact under the action of high temperature and pressure.

**Figure 4:**
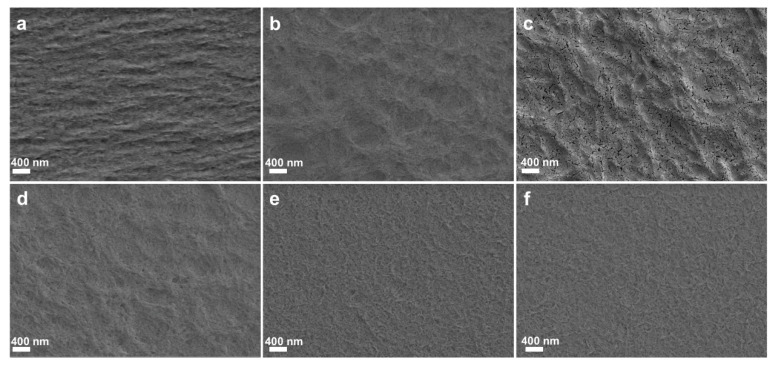
SEM images of the surfaces of (**a**) the RC0, and (**b**–**f**) RC@PU1-5 films.

SEM images of the surfaces of RC0 and RC@PU films are shown in Figure 4, and the SEM image of the surface of PU film is shown in Figure S5b. Compared with the surface of RCH (Figure S1a,b) and RC film soaked in 1 wt% PU solution and naturally air-dried (Figure S2a), the surface morphologies of RC0 and RC@PU films are greatly changed under high temperature and pressure. In particular, the number of holes and pore size on the surface of RC0 and RC@PU films decreased sharply, and all films showed a relatively flat and dense structure [40,41]. These conclusions are agreed well in the AFM diagrams of RC0 and RC@PU films (Figure S6). As shown in Figure S6a, the surface fluctuation range on RC0 is only about 0–21.4 nm. The AFM diagrams of RC@PU1-5 (Figure S6b–f) more clearly and intuitively show the difference between RC films and PU coating. It could be observed that the fluctuation ranges of RC@PU1-5 were 0–105.78 nm, 0–116.46 nm, 0–100.36 nm, 0–56.35 nm and 0–56.29 nm. In order to further characterize the average roughness of RC0 and RC@PU1-5, the root–mean–square roughness (*R*q) of RC0 and RC@PU1-5 in the figure is calculated by the analysis software (Nanoscope Analysis 3.0) in AFM, which is 3.756, 20.494, 23.061, 17.019, 9.639 and 8.592 nm, respectively. These results indicate that with an increase in PU content in the immersion solution, the surface roughness of the films increases first and then decreases, and gradually becomes stable.

Additionally, the effects of RCH surface pore size and PU particle size in 1–5 wt% PU solutions on the formation of RC@PU films were further investigated. The pore size of the RCH surface (Figure S1a,b) was analyzed, and the pore size distribution of the RCH surface was obtained (Figure S7). The average pore size of the RCH surface is 223.7 ± 4.7 nm. The newly configured 1–5 wt% PU solutions were taken for particle size test, and the particle size distribution diagram and average particle size diagram of the solution were obtained (Figure S8). The particle size of PU increases with an increase in the PU content. Moreover, when the PU content is 1 wt% and 2 wt%, and their average particle size is smaller than the average pore size of the surface of RCH, indicating that fewer PU particles can enter the hole on the surface of RCH and fit with it. The cross-section aperture of RCH is very small, typically on the scale of less than 100 nm, making it difficult for PU particles to penetrate into the RCH. These results indicate that the PU particles of RC films soaked by PU solutions are mainly distributed in the holes on the surface of RC films and above the surface of RC films. The cross-sectional SEM image of RC films soaked in 1 wt% PU solution and naturally air-dried (Figure S2b), the cross-sectional SEM image of RC@PU films obtained after hot pressing (Figure 3) and analysis mutually confirm this conclusion.

### 3.2. Physicochemical Properties of RC@PU Films

**Figure 5:**
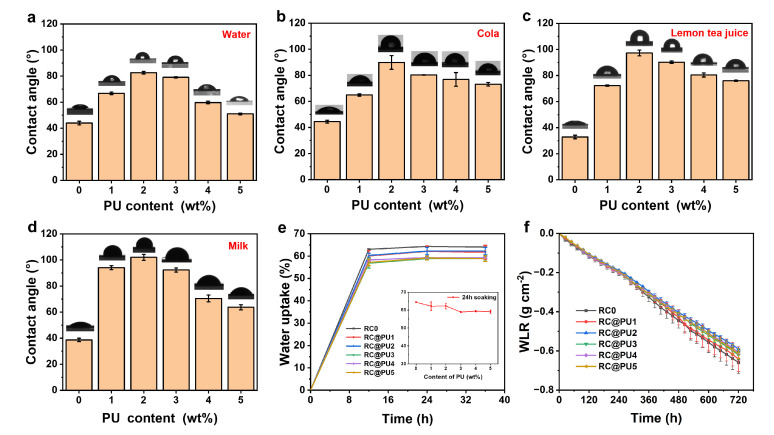
(**a**) Water, (**b**) cola, (**c**) lemon tea juice, (**d**) milk contact angle values of RC0 and RC@PU1-5 films. (**e**) Water uptake of RC0 and RC@PU1-5 films. (**f**) Weight loss rate (WLR) for devices with RC0 and RC@PU1-5 films in the water evaporation tests.

Figure 5a–d show the contact angles of RC0 and RC@PU films with water, cola, lemon tea juice and milk, respectively. In Figure 5a, the water contact angle of RC0 is 43.9°, which is attributed to the presence of a large number of hydroxyl groups on the cellulose, making the cellulose films highly hydrophilic [42]. In Figure S5c, the water contact angle of PU film is 88.2 ± 1.5°. The water contact angles of RC@PU films are higher than that of RC0, with the water contact angle of RC@PU2 reaching 82.5°, indicating that the introduction of PU coatings enhanced the hydrophobicity of the RC@PU films [3,43,44]. When in contact with drinks where water is the main component, such as cola, lemon tea, and milk, the contact angles of the RC0 film were 44.4°, 32.8° and 38.6°, respectively, all demonstrating good wettability. In contrast, the contact angles of the RC@PU2 film with these drinks were 89.8°, 97.2° and 102.0°, indicating that the RC@PU2 film is less likely to be wetted and exhibits certain anti-fouling properties against these drinks. As shown in Figure 5a–d, with an increase in PU content in the soaking solution, the contact angles between RC@PU films and the four drinks first increased and then decreased. When the PU content was between 1–2 wt%, the surface roughness of the RC@PU films gradually increased, and at the same time, hydrophobic groups, such as alkyl chains in the PU, became more prominent, resulting in an increase in the contact angles between RC@PU films and the four liquid substances within this content range.

Figure 5e shows the water absorption of RC0 and RC@PU films. Both RC0 and RC@PU films reach their peak moisture content and remain stable after soaking for about 24 h, indicating that these films have reached swelling equilibrium [45]. Moreover, the moisture content of the RC@PU films after 24 h of soaking is lower than that of RC0. Figure 5f shows the amount of water evaporation per unit area of RC0 and RC@PU films, which increases over time under natural conditions. As the evaporation time increases, the water evaporation of RC@PU films is lower than that of RC0, with the water evaporation of the RC@PU2 film being the smallest. This indicates that, under the same conditions, the RC@PU films are more effective in hindering water evaporation than RC0. This can be attributed to the PU coatings on RC@PU films, which reduce the porosity of the film surface and enhance its hydrophobicity [36]. The lower water evaporation per unit area indicates that the film has a lower water vapor transmittance. Therefore, the use of RC@PU films in food packaging can help extend shelf life [46].

**Figure 6:**
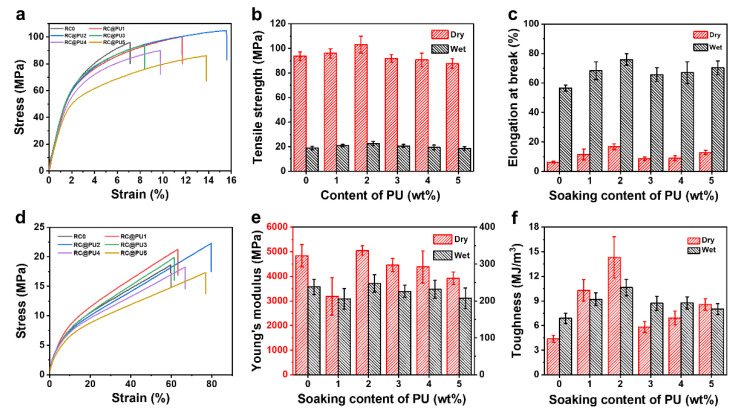
Stress–strain curves of (**a**) the dry and (**d**) wet films. The corresponding (**b**) tensile strength, (**c**) elongation at break, (**e**) Young’s modulus and (**f**) toughness of the dry and wet films.

Figure 6 shows the mechanical properties of RC0 and RC@PU films. Based on the previously discussed water absorption experiment, both RC0 and RC@PU films reach swelling equilibrium after soaking for 24 h. Therefore, the mechanical properties of the films in their wet state were evaluated by performing a tensile test after soaking the RC0 and RC@PU films in deionized water for 24 h. Figure 6a,d show the stress–strain curves of RC0 and RC@PU films under dry and wet conditions, respectively. In Figure S9a, the PU film in the dry state exhibits observable deformation before fracture, with substantial fracture occurring only when the strain reaches 990%; in Figure S9b, the PU film in the wet state also shows excellent elongation at break. Through repeated tensile test experiments, the breaking strength (Figure 6b), elongation at break (Figure 6c), elastic modulus (Figure 6e), and toughness (Figure 6f) of RC0 and RC@PU films under both dry and wet conditions were determined. In the dry state, the fracture strength of the RC0 film is 93.7 ± 3.4 MPa. After the introduction of PU coatings, the fracture strength of RC@PU1-5 films is 96.0 ± 3.8, 103.1 ± 6.9, 91.8 ± 3.2, 90.6 ± 5.5 and 87.7 ± 3.8 MPa. It can be observed that the breaking strength of RC@PU1 and RC@PU2 films is higher than that of RC0, which can be attributed to the hydrogen bonds formed between the cellulose and PU chains. However, the breaking strength of RC@PU3-5 is lower than that of RC0 because the thickness of the PU coating slightly increases with the increased PU content in the soaking solution, resulting in a reduction in film strength [36]. In addition, RC@PU2 film has better mechanical properties than other cellulosic composite films (Table S3). In the wet state, the fracture strength of RC0 film is 18.9 ± 1.3 MPa, and that of RC@PU1-5 films is 20.9 ± 0.9, 22.5 ± 1.8, 20.7 ± 1.2, 19.3 ± 1.9 and 18.6 ± 1.5 MPa, respectively. Except for RC@PU5, the wet strength of the other RC@PU films is stronger than that of RC0. These results indicate that the hydrophobicity of the RC film, enhanced by the PU coating, improves its tensile strength in the wet state.

The elongation at break of RC@PU films is greater than that of RC0 under both dry and wet conditions. This can be attributed to the improved flexibility of RC@PU films, as PU, which contains a large number of flexible chain segments, is introduced onto the surface of the RC films. Additionally, except for the RC@PU1 film, the elastic modulus of RC0 and the other RC@PU films remains in the range of 4000–5000 MPa under dry conditions and 200–250 MPa under wet conditions. This suggests that when the PU concentration in the soaking solution exceeds 1 wt%, the introduction of the PU coating does not significantly affect the elastic modulus of the RC films. Furthermore, the fracture work of RC@PU films is higher than that of RC0 under both dry and wet conditions. Notably, in the wet state, the breaking strength, elongation at break, and work at break of RC@PU2 (22.5 MPa, 75.9%, 10.6 MJ/m^3^) are significantly improved compared to RC0 (18.9 MPa, 56.5%, 6.9 MJ/m^3^). Moreover, by calculating the strength of RC0 and RC@PU1-5 under both dry and wet conditions, the moisture resistance (WR) values are obtained as 20.2%, 21.8%, 21.8%, 22.5%, 21.3%, and 21.3%, respectively, indicating that the moisture resistance of RC@PU films is higher than that of RC0. In summary, the moisture resistance and mechanical properties of the RC@PU films, obtained after soaking in a specific concentration (1–4 wt%) PU solution and hot pressing under wet conditions, are improved.

### 3.3. Biodegradability and Biocompatibility of RC@PU Films

**Figure 7:**
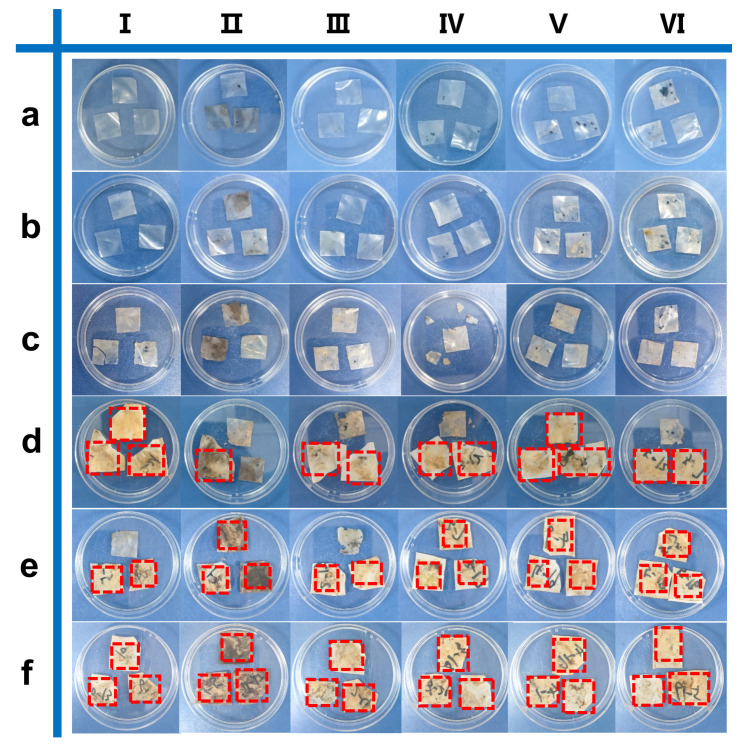
Photographs of (I) RC0 and (II–VI) RC@PU1-5 films taken at (**a**) 7, (**b**) 14, (**c**) 28, (**d**) 42, (**e**) 56, and 70 days (**f**) after starting the degradation process.

Based on the requirements for green environmental protection, excellent biodegradability is a crucial factor for the application of materials in food packaging [47]. It has previously been demonstrated that cellulose films exhibit excellent biodegradability in nature [48,49]. In this study, both the prepared RC0 and RC@PU1-5 films were sampled and buried in soil for biodegradation experiments. Figure 7 shows the macroscopic degradation of RC0 and RC@PU1-5 over a 70-day period, while Figure S10 presents the change in ambient temperature over the same period. It can be observed that during the degradation process from days 7 to 14, no significant changes were observed in any of the films. On the 28th day of degradation, a corner was missing from the edge of the film, and some yellow soil residue was visible on the membrane. As the degradation time increases, the damage to all the films gradually intensifies, with the films adhering to the nylon cloth, which may be due to the presence of microorganisms in the soil that attacked and decomposed the cellulose chains [50]. On the 56th day, only a few crumbs remained in the experimental groups of RC0 and RC@PU1-5. By the 70th day of degradation, all the experimental groups could be considered completely degraded. Additionally, the degradation time-dependent weight loss curve for RC@PU2 also indicates that it could have been fully degraded (Figure S11). These results demonstrate that RC@PU films are completely biodegradable.

The microdegradation of RC0, RC@PU2, and RC@PU5 films over 56 days was further examined using SEM (Figure S12). On the 7th and 14th days of degradation, micrometer-level damage appeared on the surface of all three film types. By the 28th day, the size of the damage increased, and shrinkage began to occur. On the 42nd day, the damage to the films became more severe, as seen in the SEM images, where overall damage is visible across the entire field of view. Notably, many microorganisms were observed on the RC@PU5 film, adhering to and corroding the surface, which indicates the involvement of microorganisms in the degradation of cellulose films in soil. By the 56th day, hundreds of micron-sized faults were visible on the membrane. In conclusion, RC@PU films demonstrate an excellent ecological closed-loop path and serve as green, degradable materials.

As shown in Figure S13, the cell viability of RC0 film is 96.65 ± 0.29%, and that of RC@PU2 film is 97.49 ± 0.81%, both higher than 90%. These data show that RC0 and RC@PU2 films have almost no cytotoxicity to L929 cells, and therefore RC@PU films demonstrate excellent biocompatibility [51].

## 4. Conclusions

This work utilized RC films as the main body and WPU as the coating material, and the RC@PU films were successfully prepared using simple and easily controlled soaking and hot-pressing steps. PU predominantly exists on the RC films as a surface coating. When the PU solution concentration was 2 wt%, the RC@PU films exhibited the best liquid wettability, which was influenced by the surface roughness and the hydrophobic alkyl chains in PU. At the same PU concentration, the obtained RC@PU films demonstrated superior mechanical properties compared to pure regenerated cellulose films, both in dry (103.1 MPa, 16.8%, 14.3 MJ/m^3^) and wet states (22.5 MPa, 75.9%, 10.6 MJ/m^3^). Therefore, RC@PU films with excellent mechanical properties can effectively protect inclusions in practical applications and reduce the impact of external liquids (such as milk and cola) on these inclusions. Biodegradation experiments show that RC@PU films can be completely degraded in soil under natural environmental conditions. This work provides a simple, environmentally friendly, and effective approach to preparing regenerated cellulose films with high strength and excellent biodegradability, demonstrating significant potential for applications in green packaging materials.

## Data Availability

The data presented in this study are available on request from the corresponding author.

## Supplementary Materials

The following supporting information can be downloaded at: https://www.mdpi.com/article/10.3390/polym17070890/s1, Table S1. Decomposition temperature of RC0 and RC@PU1-5 films. Table S2. The element compositions of RC@PU1 film measured by EDS in Figure S3.; Table S3. Mechanical properties of cellulosic films with various composites. Figure S1. SEM images of (a,b) the surface and (c,d) cross-section of the RCH after freezing-dried.; Figure S2. SEM images of (a) the surface and (b) cross-section of RC@PU1 film; Figure S3. SEM image and the corresponding point scanning of element distribution spectra of RC@PU1 film; Figure S4. Thickness of PU coating measured from SEM images of the cross-section of RC@PU films; Figure S5. SEM images of (a) the cross-section and (b) surface of PU films. (c) Water contact angle of PU films; Figure S6. AFM images of the surface of (a) RC0 and (b–f) RC@PU1-5 films; Figure S7. Size distribution measured from SEM image of the surface of RCH; Figure S8. (a) Size distribution and (b) the average particle size of 1~5 wt% PU solutions; Figure S9. Stress–strain curves of (a) the dry and (b) wet PU films; Figure S10. Change of ambient temperature within 70 days in the biodegradation experiment; Figure S11. Dependence of the weight loss on the degradation time for the RC@PU2 film degraded in soil of the natural environment; Figure S12. SEM images of the surface of the RC0, RC@PU2 and RC@PU5 films degraded in soil of the natural environment for 7, 14, 28, 42 and 56 days.; Figure S13. Cell viability of L929 cells with the RC0 and RC@PU2 films. References [52,53,54,55,56,57] are cited in Supplementary Materials.
