# Supplementary material for: Regenerated Cellulose Films Coated with Waterborne Polyurethane with Enhanced Mechanical Properties
Source: Polymers (Basel). 2025 Mar 26;17(7):890. doi: 10.3390/polym17070890 (PMC11991312; doi:10.3390/polym17070890)
Supplement: Supplementary file 1 [file polymers-17-00890-s001.zip › polymers-3514673-supplementary.pdf]

Supplementary Materials

Regenerated cellulose films coated with waterborne polyurethane with enhanced mechanical properties

*Renxiang Xiong, Jinping Zhou**

Hubei Engineering Center of Natural Polymers-based Medical Materials, College of Chemistry and Molecular Sciences, Wuhan University, Wuhan 430072, China

Corresponding author: zhoujp325@whu.edu.cn

The supporting information contains:

Table S1-S3

Figures S1-S13

Table S1. Decomposition temperature of RC0 and RC@PU1-5 films.

Sample	RC0	RC@PU1	RC@PU2	RC@PU3	RC@PU4	RC@PU5
T_d (°C)	342.8±0.5	343.4±0.2	344.9±0.4	345.9±0.3	345.4±0.7	344.9±0.9

Table S2. The element compositions of RC@PU1 film measured by EDS in **Figure S3**.

Fig	C (wt%)	C_σ (wt%)	N (wt%)	N_σ (wt%)	O (wt%)	O_σ (wt%)
S3b	80.23	0.44	1.07	0.20	18.70	0.20
S3c	86.36	0.26	0.48	0.19	13.16	0.20
S3d	85.86	0.20	0.00	0.18	14.14	0.20

Table S3. Mechanical properties of cellulosic films with various composites.

Typical sample	Cellulosic materials	Composites	Tensile strength (MPa)	Elongation at break (%)	Ref.
RC@PU2	Cellulose	PU	96.2-110.0	14.8-18.7	This work
C10-CNF-C10	CNF	Cellulose esters with different side chain lengths (C6–C18)	82-102	11-14	[1]
RC-20G-15Z	Cellulose	Zein	35-52	2.6-5.7	[2]
TOCNs/TOWFs-C	CNF	TEMPO-oxidized wood fibers	75-80	10.49-15.3	[3]
WCF-0.4%	Cellulose	Ca^{2+}	26.8-78.5	4.3-11.5	[4]
RC6	Cellulose	SiO_2	40.1-77.2	3-14	[5]
Cellulose/ (1 wt%) Fe_3O_4	Cellulose	Iron oxide	50-57	5.8-9.4	[6]

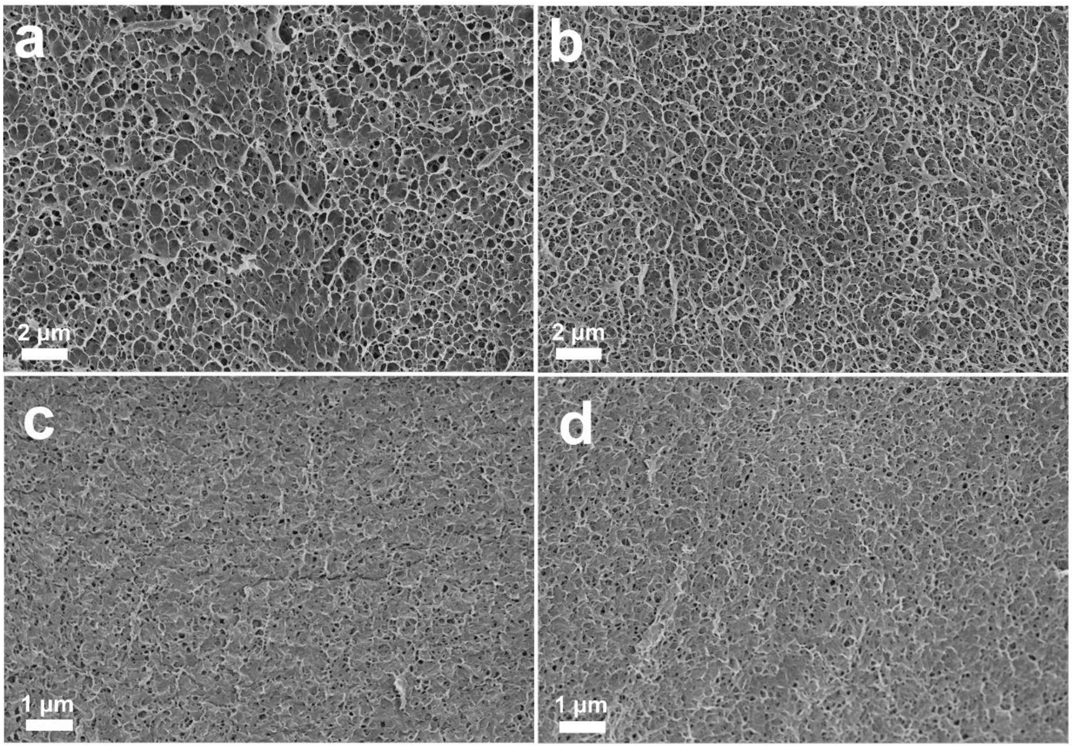

Figure S1. SEM images of (a,b) the surface and (c,d) cross-section of the RCH after freezing-dried.

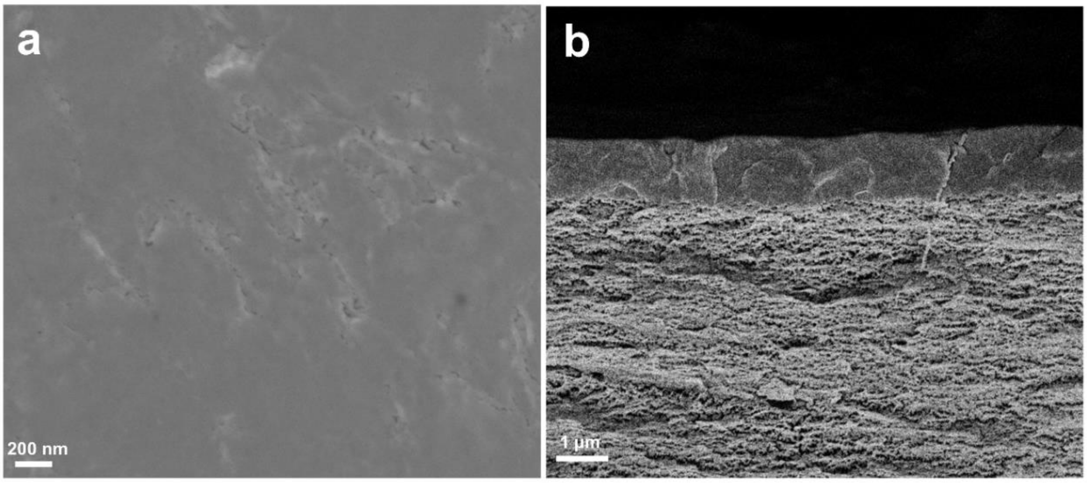

Figure S2. SEM images of (a) the surface and (b) cross-section of RC@PU1 film.

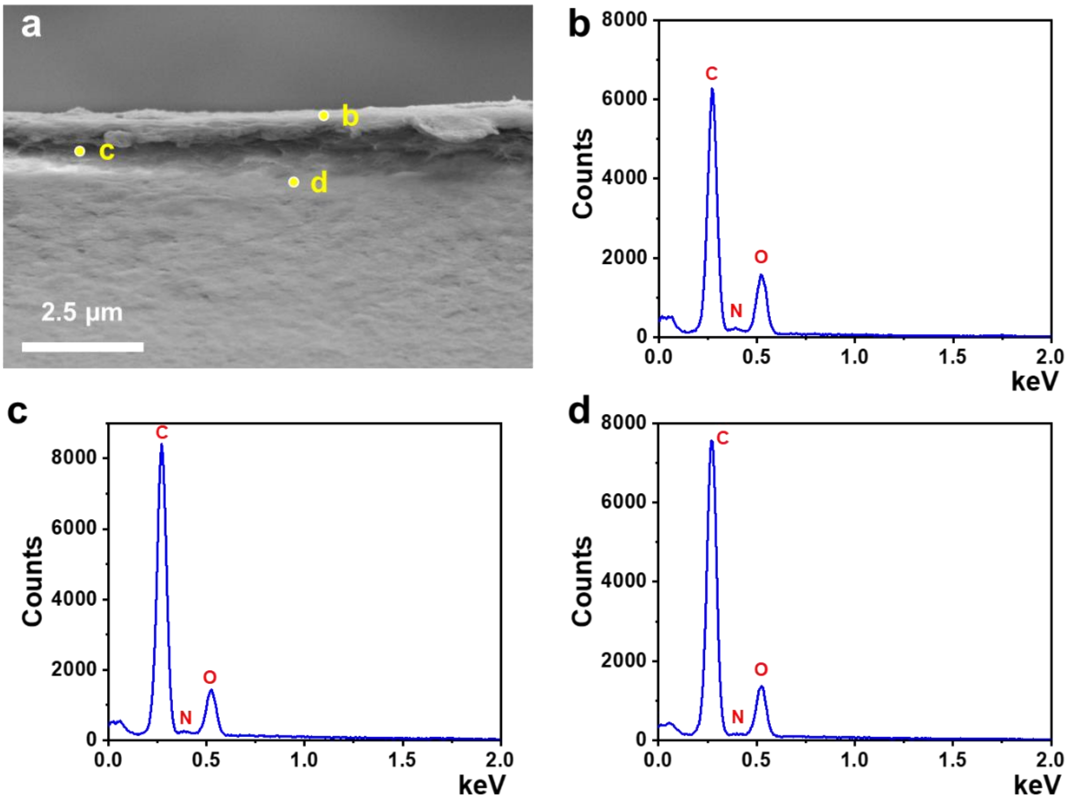

Figure S3. SEM image and the corresponding point scanning of element distribution spectra of RC@PU1 film.

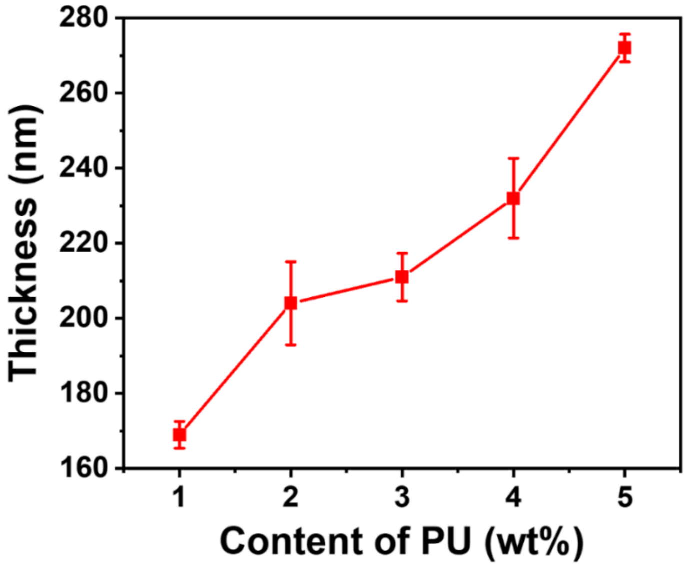

Figure S4. Thickness of PU coating measured from SEM images of the cross-section of RC@PU films.

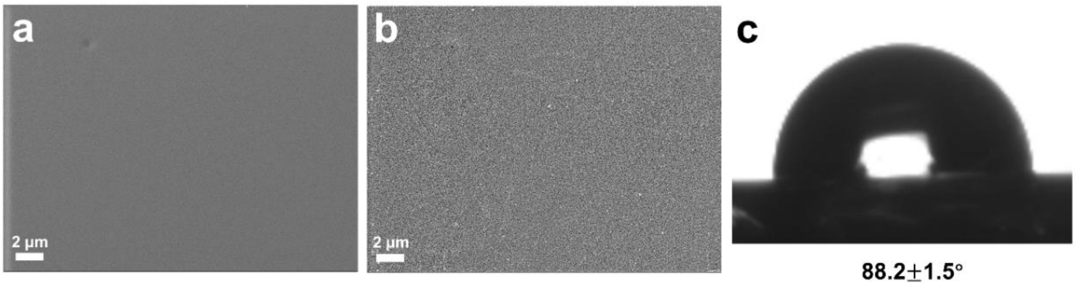

Figure S5. SEM images of (a) the cross-section and (b) surface of PU films. (c) Water contact angle of PU films.

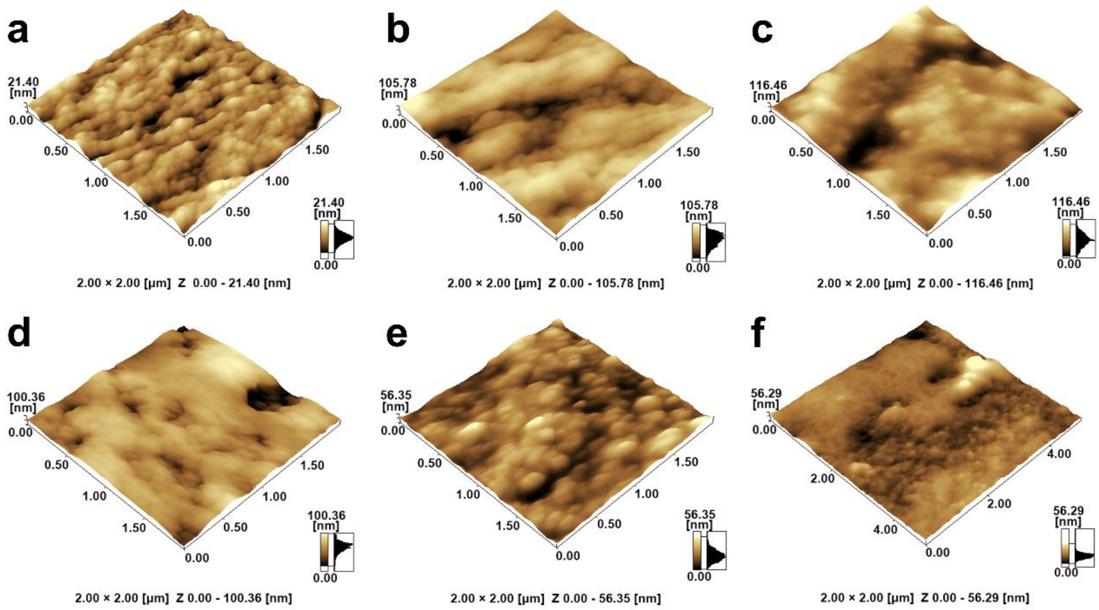

Figure S6. AFM images of the surface of (a) RC0 and (b-f) RC@PU1-5 films.

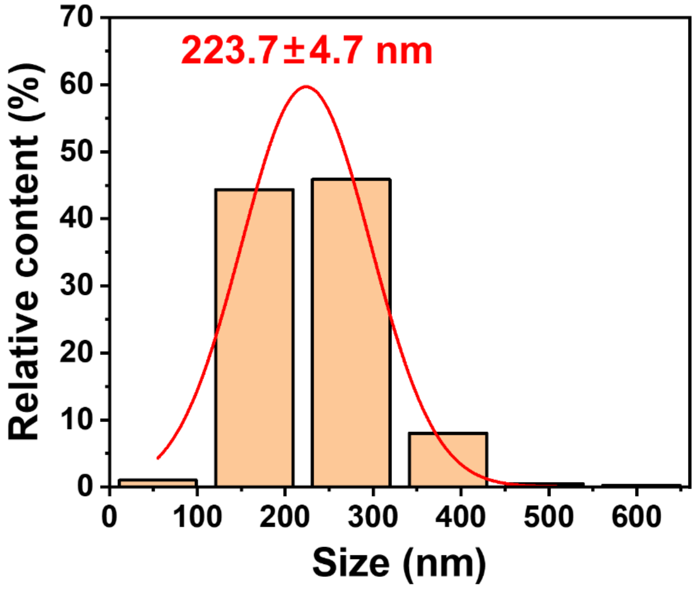

Figure S7. Size distribution measured from SEM image of the surface of RCH.

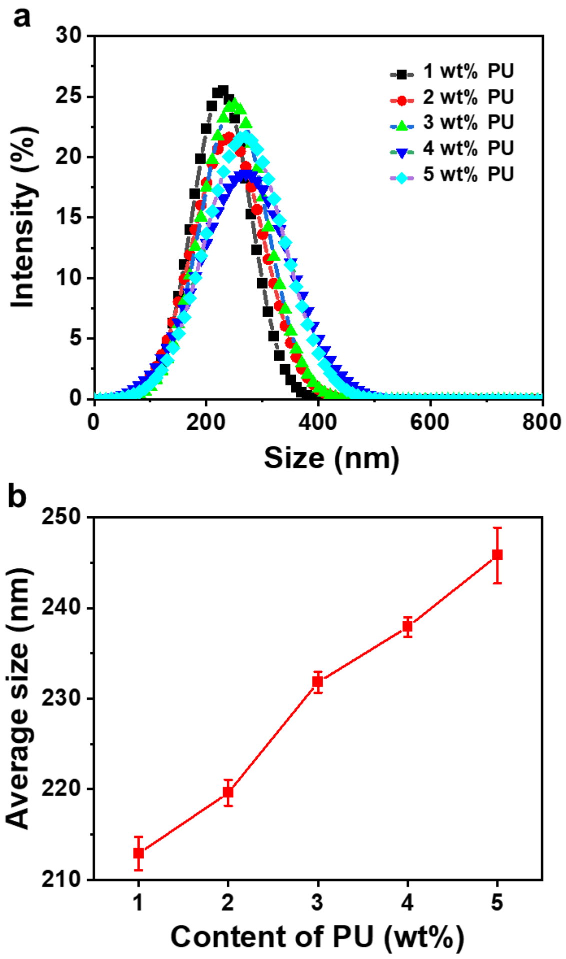

Figure S8. (a) Size distribution and (b) the average particle size of 1~5 wt% PU solutions.

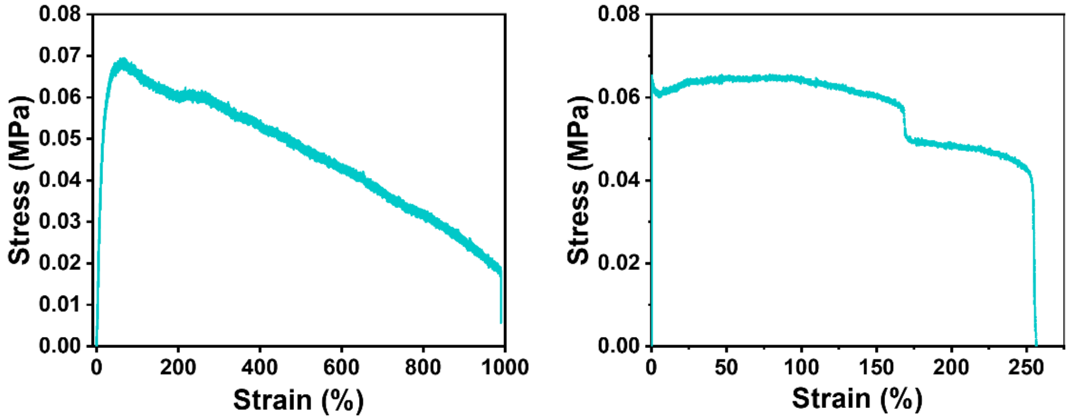

Figure S9. Stress–strain curves of (a) the dry and (b) wet PU films.

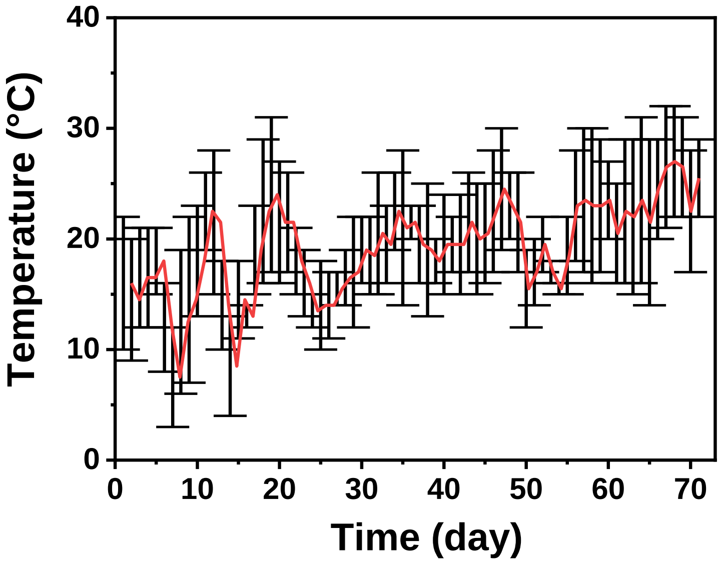

Figure S10. Change of ambient temperature within 70 days in the biodegradation experiment.

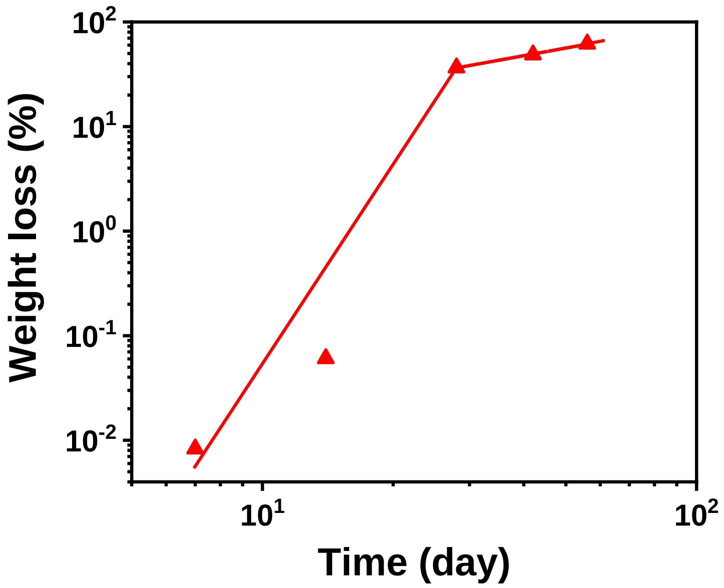

Figure S11. Dependence of the weight loss on the degradation time for the RC@PU2 film degraded in soil of the natural environment.

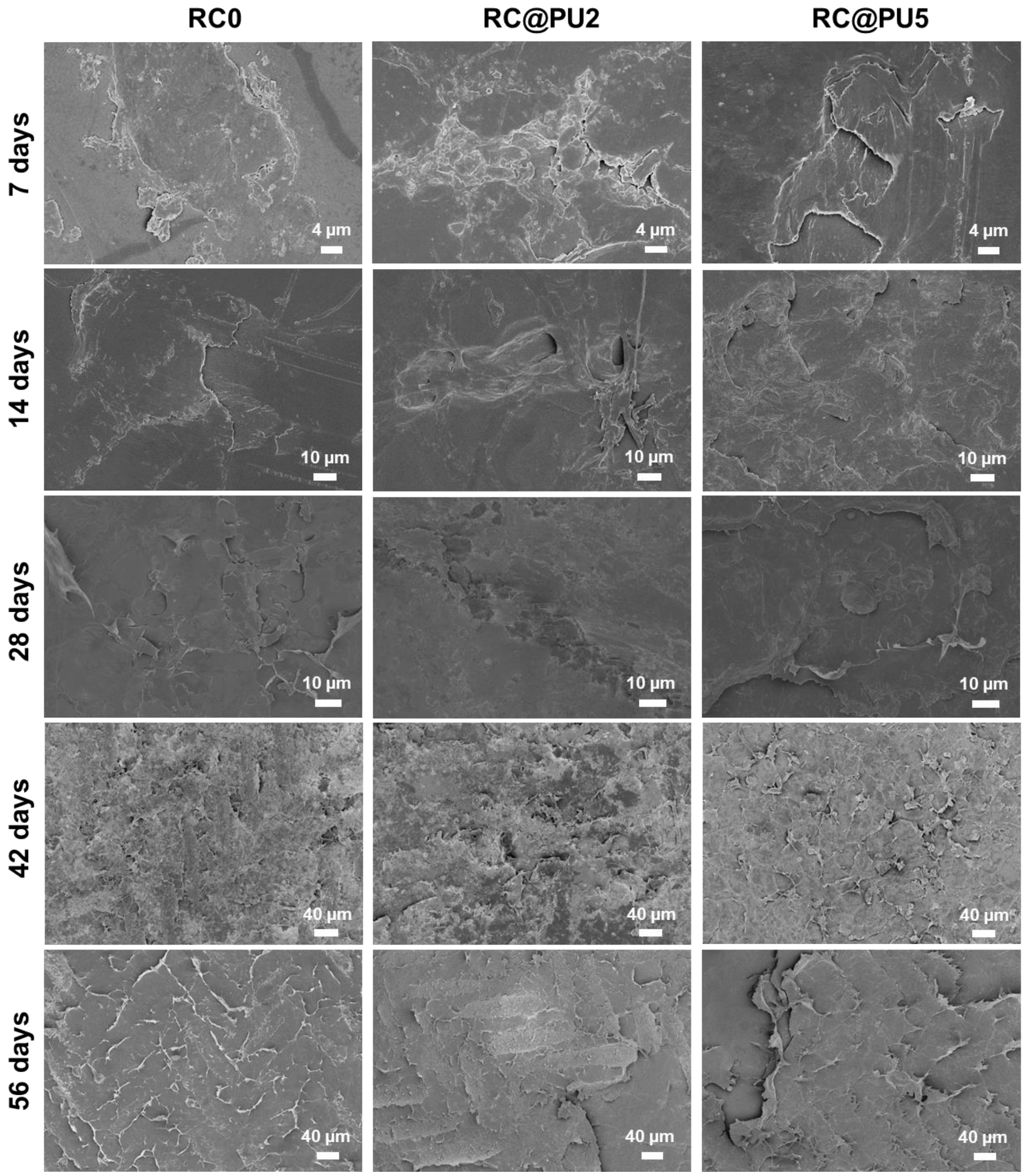

Figure S12. SEM images of the surface of the RC0, RC@PU2 and RC@PU5 films degraded in soil of the natural environment for 7, 14, 28, 42 and 56 days.

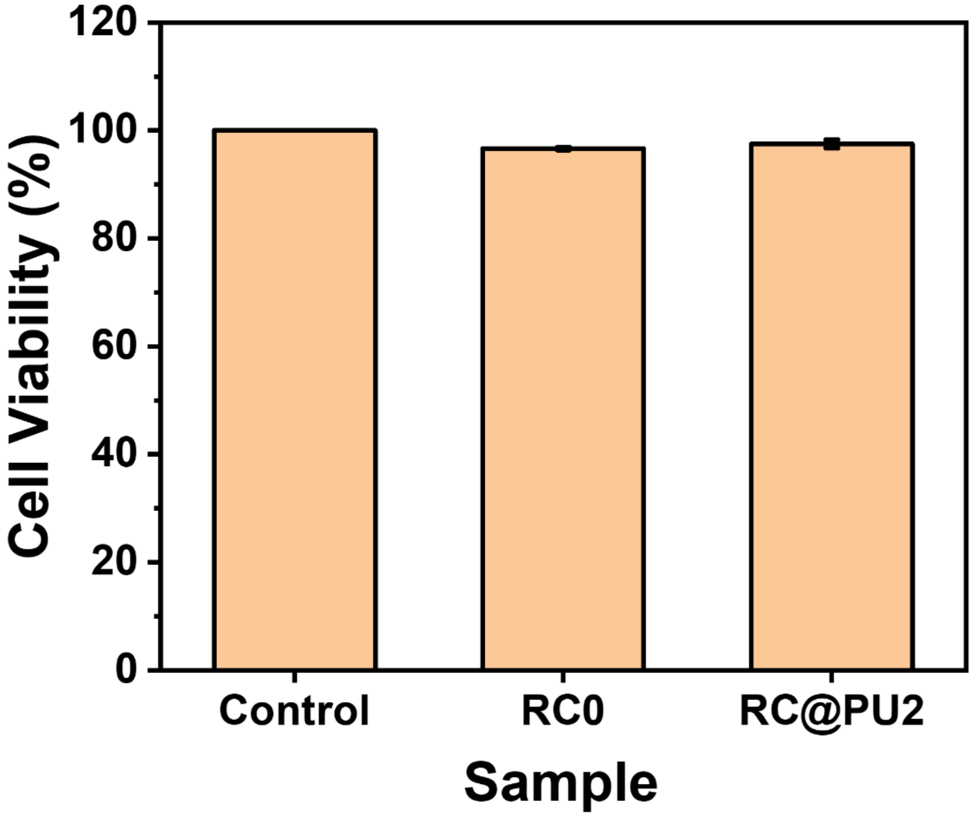

Figure S13. Cell viability of L929 cells with the RC0 and RC@PU2 films.

References

1. Willberg-Keyriläinen, P.; Vartiainen, J.; Pelto, J.; Ropponen, J. Hydrophobization and smoothing of cellulose nanofibril films by cellulose ester coatings. *Carbohydr. Polym.* **2017**, *170*, 160-165.
2. Chu, Y.; Popovich, C.; Wang, Y. Heat sealable regenerated cellulose films enabled by zein coating for sustainable food packaging. *Composites Part C,* **2023**, *12*, 100390.
3. Yang, W.; Jiao, L.; Liu, W.; Dai, H. Manufacture of highly transparent and hazy cellulose nanofibril films via coating TEMPO-oxidized wood fibers, *Nanomaterials,* **2019**, *9,* 107.
4. Gao, Y.; Huang, C.; Ge, D.; Liao, Y.; Chen, Y.; Li, S.; Yu, H.-Y. Highly efficient dissolution and reinforcement mechanism of robust and transparent cellulose films for smart packaging. *Int. J. Biol. Macromol.* **2024**, *254*, 128046.
5. Zhu, Y.; Wang, T.; Dai, Y.; Wang, Y.; Ding, Y.; Zhang, L. Surface engineering of regenerated cellulose nanocomposite films with high strength, ultraviolet resistance, and a hydrophobic surface. *Polymers* **2023**, *15* (6), 1427.
6. Yadav, M. Study on thermal and mechanical properties of cellulose/iron oxide bionanocomposites film. *Compos. Commun.* **2018**, *10*, 1-5.
